# Sweet's syndrome – a comprehensive review of an acute febrile neutrophilic dermatosis

**DOI:** 10.1186/1750-1172-2-34

**Published:** 2007-07-26

**Authors:** Philip R Cohen

**Affiliations:** 1 University of Houston Health Center, Houston, Texas, USA; 2 The Department of Dermatology, University of Texas-Houston Medical School, Houston, Texas, USA

## Abstract

Sweet's syndrome (the eponym for acute febrile neutrophilic dermatosis) is characterized by a constellation of clinical symptoms, physical features, and pathologic findings which include fever, neutrophilia, tender erythematous skin lesions (papules, nodules, and plaques), and a diffuse infiltrate consisting predominantly of mature neutrophils that are typically located in the upper dermis. Several hundreds cases of Sweet's syndrome have been published. Sweet's syndrome presents in three clinical settings: classical (or idiopathic), malignancy-associated, and drug-induced. Classical Sweet's syndrome (CSS) usually presents in women between the age of 30 to 50 years, it is often preceded by an upper respiratory tract infection and may be associated with inflammatory bowel disease and pregnancy. Approximately one-third of patients with CSS experience recurrence of the dermatosis. The malignancy-associated Sweet's syndrome (MASS) can occur as a paraneoplastic syndrome in patients with an established cancer or individuals whose Sweet's syndrome-related hematologic dyscrasia or solid tumor was previously undiscovered; MASS is most commonly related to acute myelogenous leukemia. The dermatosis can precede, follow, or appear concurrent with the diagnosis of the patient's cancer. Hence, MASS can be the cutaneous harbinger of either an undiagnosed visceral malignancy in a previously cancer-free individual or an unsuspected cancer recurrence in an oncology patient. Drug-induced Sweet's syndrome (DISS) most commonly occurs in patients who have been treated with granulocyte-colony stimulating factor, however, other medications may also be associated with DISS. The pathogenesis of Sweet's syndrome may be multifactorial and still remains to be definitively established. Clinical and laboratory evidence suggests that cytokines have an etiologic role. Systemic corticosteroids are the therapeutic gold standard for Sweet's syndrome. After initiation of treatment with systemic corticosteroids, there is a prompt response consisting of dramatic improvement of both the dermatosis-related symptoms and skin lesions. Topical application of high potency corticosteroids or intralesional corticosteroids may be efficacious for treating localized lesions. Other first-line oral systemic agents are potassium iodide and colchicine. Second-line oral systemic agents include indomethacin, clofazimine, cyclosporine, and dapsone. The symptoms and lesions of Sweet's syndrome may resolved spontaneously, without any therapeutic intervention; however, recurrence may follow either spontaneous remission or therapy-induced clinical resolution.

## Disease name and synonyms

Sweet's syndrome

Acute febrile neutrophilic dermatosis

Gomm-Button disease

## Introduction

The syndrome was originally described by Dr. Robert Douglas Sweet in the August-September 1964 issue of the British Journal of Dermatology as an "acute febrile neutrophilic dermatosis" [1-10]. This seminal paper summarizes the cardinal features of "a distinctive and fairly severe illness" that he had encountered in eight women during the 15-year period from 1949 to 1964. In his disease defining report, Dr. Sweet commented that "the condition was known in my department as the Gomm-Button disease, a title some may still prefer to that which heads this paper;" this nomenclature was "in eponymous honor of the first two patients" with the disease in Dr. Sweet's department [8]. Subsequently, Dr. Sweet recommended that the name of the condition remain descriptive; however, in spite of his suggestion, 'Sweet's syndrome' has become the established eponym for this acute febrile neutrophilic dermatosis [1-10]. Several hundreds of reports of Sweet's syndrome patients have since been published [11-435].

### Definition and diagnostic criteria

Sweet's syndrome can present in several clinical settings: classical (or idiopathic) Sweet's syndrome, malignancy-associated Sweet's syndrome, and drug-induced Sweet's syndrome.

#### Classical Sweet's syndrome

**Figure 1:**
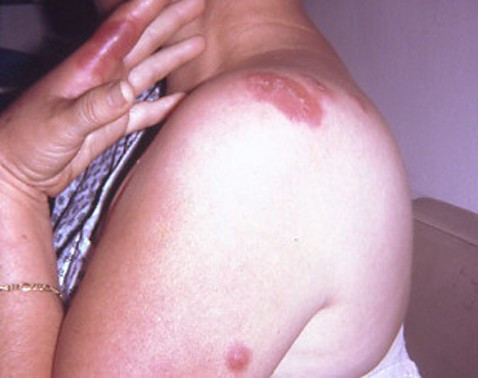
Sweet's syndrome skin lesions in a woman with classical Sweet's syndrome. Cutaneous lesions of classical Sweet's syndrome on the left hand, left proximal arm and left shoulder in a 48-year-old woman with pyrexia, neutropenia, and a recent respiratory tract infection. (From **[10] **Cohen PR, Almeida L, Kurzrock R: Acute febrile neutrophilic dermatosis. Am Fam Physician 1989;39(3):199–204. *Copyright 1989, Reprinted with permission from Academy of American Family Physicians, Leewood, Kansas*.)

**Figure 2:**
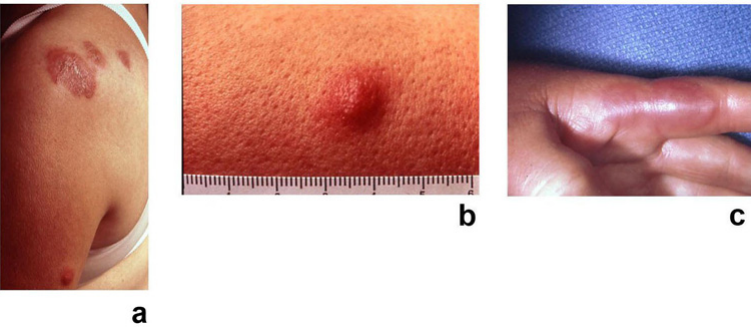
**(a, b, and c)**. Tender, red Sweet's syndrome lesions in a woman with the classical form of the dermatosis. A closer view of the Sweet's syndrome lesions from the woman in Figure 1. The skin lesions improved rapidly after corticosteroid therapy was initiated. There is an erythematous plaque, 5 cm in diameter, with a pseudovesicular appearance on the left shoulder of the patient (a). A nodular lesion, 1 cm in diameter, is present on the lateral left arm (b). Painful, erythematous, pseudovesicular plaques of acute febrile neutrophilic dermatosis are present on the left hand (c). (From **[10] **Cohen PR, Almeida L, Kurzrock R: Acute febrile neutrophilic dermatosis. Am Fam Physician 1989;39(3):199–204. *Copyright 1989, Reprinted with permission from Academy of American Family Physicians, Leewood, Kansas*.)

Classical Sweet's syndrome is characterized by a constellation of clinical symptoms, physical features, and pathologic findings which include pyrexia, elevated neutrophil count, tender erythematous skin lesions (papules, nodules, and plaques), and a diffuse infiltrate consisting predominantly of mature neutrophils typically located in the upper dermis. The symptoms and clinical manifestations typically respond promptly after initiation of systemic corticosteroid therapy (Figures 1 and 2).

The diagnostic criteria for classical Sweet's syndrome were originally proposed by Su and Liu [11] in 1986. They were modified by von den Driesch [12] in 1994 (Table 1) [13,14]. Additional cases of Sweet's syndrome continue to appear in the medical literature since Sweet's original paper [1-435].

**Table 1:** Diagnostic criteria for classical Sweet's syndrome versus drug-induced Sweet's syndrome

Classical^a^	Drug-induced^b^
(1) Abrupt onset of painful erythematous plaques or nodules	(A) Abrupt onset of painful erythematous plaques or nodules
(2) Histopathologic evidence of a dense neutrophilic infiltrate without evidence of leukocytoclastic vasculitis	(B) Histopathologic evidence of a dense neutrophilic infiltrate without evidence of leukocytoclastic vasculitis
(3) Pyrexia >38°C	(C) Pyrexia >38°C
(4) Association with an underlying hematologic or visceral malignancy, inflammatory disease, or pregnancy, OR preceded by an upper respiratory or gastrointestinal infection or vaccination	(D) Temporal relationship between drug ingestion and clinical presentation, OR temporally-related recurrence after oral challenge
(5) Excellent response to treatment with systemic corticosteroids or potassium iodide	(E) Temporally-related resolution of lesions after drug withdrawal or treatment with systemic corticosteroids
(6) Abnormal laboratory values at presentation (three of four): erythrocyte sedimentation rate >20 mm/hr; positive C-reactive protein; >8,000 leukocytes; >70% neutrophils	

^a^The presence of both major criteria (1 and 2), and two of the four minor criteria (3, 4, 5, and 6) is required in order to establish the diagnosis of classical Sweet's syndrome; the patients with malignancy-associated Sweet's syndrome are included with the patients with classical Sweet's syndrome in this list of diagnostic criteria.

^b^All five criteria (A, B, C, D, and E) are required for the diagnosis of drug-induced Sweet's syndrome.

Source **[13]**: Adapted with permission from Walker DC, Cohen PR: Trimethoprim-sulfamethoxazole-associated acute febrile neutrophilic dermatosis: case report and review of drug induced Sweet's syndrome. J Am Acad Dermatol 1996;34:918–923. *Copyright 1996, Reprinted with permission from the American Academy of Dermatology, Inc., Elsevier Ltd, Oxford, United Kingdom*.

#### Malignancy-associated Sweet's syndrome

Shapiro *et al *[24] reported the first patient with solid tumor-associated Sweet's syndrome, a 58-year-old man with testicular carcinoma, in 1971. However, prior to Dr. Sweet's 1964 publication, Costello *et al *[29] described a 16-year-old girl with acute myelogenous leukemia and recurrent cutaneous lesions of variable morphologies in 1955. Retrospectively, several authors have acknowledged that Costello *et al*'s patient represents the first report of malignancy-associated Sweet's syndrome. It was not until 18 years later, in 1973, that Matta and Kurban [25] reported two women whose biopsy-confirmed Sweet's syndrome lesions were the presenting manifestation of their previously unsuspected acute leukemia.

Malignancy-associated Sweet's syndrome was initially included as a subset of classical Sweet's syndrome. However, since many of the cases of Sweet's syndrome are cancer-related, several authors have chosen to distinguish between the classical form and the malignancy-associated form of this condition. The onset of Sweet's syndrome can precede, follow, or appear concurrent with the diagnosis of the patient's neoplasm. Indeed, the dermatosis can be the cutaneous harbinger of either an undiagnosed visceral malignancy in a previously cancer-free individual or an unsuspected cancer recurrence in an oncology patient.

#### Drug-induced Sweet's syndrome

Su and Liu [11] reported the first patient with drug-induced Sweet's syndrome in 1986; the associated medication was trimethoprim-sulfamethoxazole. A decade later, criteria for drug-induced Sweet's syndrome were established by Walker and Cohen [13]. The most frequently implicated drug is granulocyte-colony stimulating factor. However, several other medications – albiet less often – have been observed to promote the development of Sweet's syndrome.

Diagnostic criteria for drug-induced Sweet's syndrome were presented by Walker and Cohen [13] in 1996 (Table 1).

## Epidemiology

The distribution of Sweet's syndrome cases is worldwide and there is no racial predilection (Table 2) [1,2,12,13,15-20,30,31].

**Table 2:** Clinical features in patients with Sweet's syndrome

	**Clinical Form**			
***Characteristic***	***Classical* ^a^**	***Hematologic malignancy* ^a^**	***Solid tumor* ^a^**	***Drug-induced* ^b^**

***Epidemiology***				
Women	80	50	59	71
Prior upper respiratory tract infection	75–90	16	20	21
Recurrence^c^	30	69	41	67
***Clinical symptoms***				
Fever^d^	80–90	88	79	100
Musculoskeletal involvement	12–56	26	34	21
Ocular involvement	17–72	7	15	21
***Lesion location***				
Upper extremities	80	89	97	71
Head and neck	50	63	52	43
Trunk and back	30	42	33	50
Lower extremities	Infrequent	49	48	36
Oral mucous membranes	2	12	3	7
***Laboratory findings***				
Neutrophilia^e^	80	47	60	38
Elevated erythrocyte sedimentation rate^f^	90	100	95	100
Anemia^g^	Infrequent	82	83	100
Abnormal platelet count^h^	Infrequent	68	50	50
Abnormal renal function^i^	11–50	15	7	0

^a^Percentages for classical, hematologic malignancy, and solid tumor associated Sweet's syndrome adapted with permission from Cohen PR, Kurzrock R: Sweet's syndrome and cancer. Clin Dermatol 1993;11:149–157 **[15]**. *Copyright 1993, Reprinted with permission from Elsevier Ltd, Oxford, United Kingdom*.

^b^Percentages for drug-induced Sweet's syndrome adapted with permission from Walker DC, Cohen PR: Trimethoprim-sulfamethoxazole-associated acute febrile neutrophilic dermatosis: case report and review of drug induced Sweet's syndrome. J Am Acad Dermatol 1996;34:918–923 **[13]**. *Copyright 1996, Reprinted with permission from the American Academy of Dermatology, Inc., Elsevier Ltd, Oxford, United Kingdom*.

^c^Recurrence following oral rechallenge testing in the patients with drug-induced Sweet's syndrome.

^d^Temperature greater than 38°C.

^e^Neutrophil count greater than 6,000 cells/ul.

^f^Erythrocyte sedimentation rate greater than 20 mm/hr.

^g^Hemoglobin less than 13 g/dl in men and less than 12 g/dl in women.

^h^Platelet count less than 150,000/ul or greater than 500,000/ul.

^i^This includes hematuria, proteinuria, and renal insufficiency.

***Classical or idiopathic Sweet's syndrome ***predominantly affects in women. It may be associated with infection (upper respiratory tract or gastrointestinal tract), inflammatory bowel disease, or pregnancy [13,15]. Recurrence of the dermatosis is noted in approximately one-third of individuals.

The initial episode of classical Sweet's syndrome most frequently occurs between the ages of 30 to 60 years. However, classical Sweet's syndrome has been reported in children (as young as 7 weeks of age) and younger adults [32-48]. Brothers who developed the dermatosis at 10 and 15 days of age are the youngest Sweet's syndrome patients reported [46].

***Malignancy-associated Sweet's syndrome ***has been published as descriptions of individual oncology patients with Sweet's syndrome, observations from small series of Sweet's syndrome patients with cancer, and retrospective reviews in which the features from multiple Sweet's syndrome patients (some with malignancy) are summarized. In 1993, Cohen and Kurzrock [15] reviewed and combined the data from 15 studies of patients with Sweet's syndrome studies (each containing between ten to 48 individuals) in order to more accurately define the incidence of malignancy-associated Sweet's syndrome. They found that 21 percent of the patients with Sweet's syndrome (96 of 448 individuals) had either a hematologic malignancy or a solid tumor.

Several investigators consider it appropriate to distinguish malignancy-associated Sweet's syndrome from the classical form of this disease. Malignancy-associated Sweet's syndrome occurs as frequently in men as in women. Also, it is less often preceded by an upper respiratory tract infection. In addition, the onset or recurrence of many of the cases of malignancy-associated Sweet's syndrome is temporally associated with the discovery or relapse of cancer. Specifically, in these individuals, either the new discovery of an unsuspected neoplasm in a patient in whom cancer has not previously been diagnosed or the recurrence of malignancy in a patient with a previously established cancer is temporally associated with the appearance of the dermatosis [15,49-60].

Malignancy-associated Sweet's syndrome is most often associated with acute myelogenous leukemia [61,62]. However, in patients with hematologic disorders, Sweet's syndrome can occur in one or more of the following forms: a paraneoplastic syndrome, a drug-induced dermatosis, or a condition whose skin lesions concurrently demonstrate leukemia cutis. Carcinomas of the genitourinary organs, breast, and gastrointestinal tract are the most frequently occurring cancers in Sweet's syndrome patients with dermatosis-related solid tumors [1,2,63-66].

### Drug-induced Sweet's syndrome

**Figure 3:**
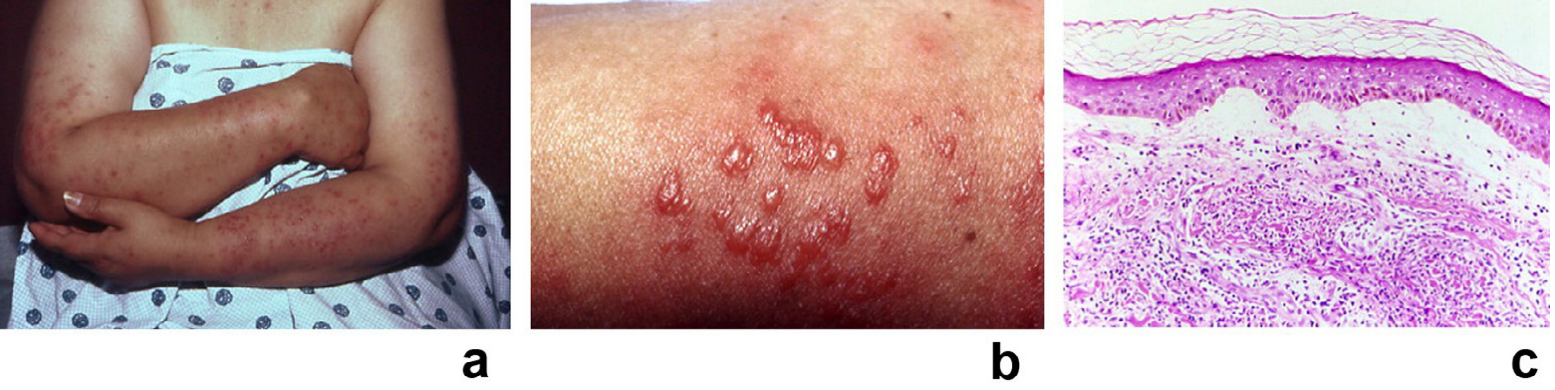
**(a, b, and c)**. Drug-induced Sweet's syndrome with photodistributed skin lesions. Photodistributed drug-induced Sweet's syndrome in a 50-year-old woman with trimethoprim-sulfamethoxazole-associated acute febrile neutrophilic dermatosis. Distant (a) and closer (b) views of Sweet's syndrome lesions located on the sun-exposed areas of the arms, hands, and upper chest are shown. The biopsy specimen (c) shows a confluent neutrophilic infiltrate in the reticular dermis and edema in the papillary dermis (hematoxylin and eosin, × 50). (From **[13] **Walker DC, Cohen PR: Trimethoprim-sulfamethoxazole-associated acute febrile neutrophilic dermatosis: case report and review of drug-induced Sweet's syndrome. J Am Acad Dermatol 1996;34:918–923. *Copyright 1996, Reprinted with permission from the American Academy of Dermatology, Inc, Elsevier Ltd, Oxford, United Kingdom*.)

Several medications have been associated with the subsequent development of drug-induced Sweet's syndrome (Table 3) [1,2,11,13,17,39,41,67-124,398,401,404,417,426,435] (Figure 3). However, the drug-induced variant of the dermatosis has most frequently been observed in patients following the administration of granulocyte-colony stimulating factor. Recurrence of the dermatosis is often noted when the patient is rechallenged with the associated drug. However, once the causative agent has been discontinued, the disease manifestations frequently improve.

**Table 3:** Medications associated with drug-induced Sweet's syndrome [a-c]

Antibiotics	Minocycline [110-112]
	Nitrofurantoin [113]
	Norfloxacin [114]
	Ofloxacin [115]
	Quinupristin/dalfopristin [118]
	Trimethoprim-sulfamethoxazole [11,13]
Antiepileptics	Carbemazepine [17]
	Diazepam [86]
Antihuman immunodeficiency virus drugs	Abacavir (synthetic carbocyclic nucleoside analogue) [69]
Antihypertensives	Hydralazine [107]
Antineoplastics	Bortezomib [d] [78-79]
	Imatinib mesylate [e] [108,109,401]
	Lenalidomide [f] [426]
Antipsychotics	Clozapine [82]
Antithyroid hormone synthesis drugs	Propylthiouracil [117]
Colony stimulating factors	Granulocyte-colony stimulating factor [39,41,89-105,398]
	Granulocyte-macrophage-colony stimulating factor [105,106]
	Pegfilgrastim [g] [116]
Contraceptives [83]	Levonorgestrel/ethinyl estradiol (Triphasil) [84]
	Levonorgestrel-releasing intrauterine system (Mirena) [85]
Diuretics	Furosemide [88]
Nonsteroidal anti-inflammatory agents	Celecoxib [80]
	Diclofenac [87]
Retinoids	All-trans retinoic acid [70-77,417]
	13-cis-retinoic acid [81,404]

[a] The possibility of acyclovir-induced Sweet's syndrome cannot be completely ruled out in a 13-year-old girl with systemic lupus erythematosus (that had been diagnosed 3 months earlier and was being treated with oral prednisolone and azathioprine) whose Sweet's syndrome lesions appeared 5 days after beginning intravenous acyclovir treatment for a herpes zoster infection [119].

[b] Photodistributed neutrophilic dermatosis with overlapping features of Sweet's syndrome, acute generalized exanthematous pustulosis and sterile neutrophilic folliculitis with perifollicular vasculopathy developed in a patient who had received antidepressant (amoxapine and citalopram) and anxiolytic (perphenazine) therapy [120].

[c] Additional reports have attributed Sweet's syndrome to medications (minocycline [121], furosemide [122], and hydralazine [123,124]); however, they did not fulfill the criteria proposed by Walker and Cohen [13] for drug-induced Sweet's syndrome.

[d] This drug is a reversible inhibitor of the chymotrypsin-like activity of the 26S proteasome in mammalian cells. It is indicated for the treatment of multiple myeloma patients.

[e] This drug is a protein-tyrosine kinase inhibitor that inhibits the bcr-abl tyrosine kinase, the constitutive abnormal tyrosine kinase created by the Philadelphia chromosome abnormality in chronic myeloid leukemia.

[f] This drug is an amino-substituted analogue of thalidomide. It is an immunomodulatory agent with anti-angiogenic and antineoplastic properties. It is indicated for the treatment of: (1) multiple myeloma and (2) transfusion dependent anemia due to low-risk or intermediate-risk myelodysplastic syndromes.

[g] This drug is a covalent conjugate of recombinant methionyl human granulocyte-colony stimulating factor (Filgrastim) and monomethoxypolyethylene.

## Clinical description

### Symptoms

Sweet's syndrome patients may appear dramatically ill. Fever is the most frequent symptom. Indeed, the skin eruption of Sweet's syndrome is usually accompanied by fever and leukocytosis. However, the cutaneous manifestations of the disease may be preceded by several days to weeks of fever. Alternatively, pyrexia can be concurrently present throughout the entire episode of the dermatosis. Also, in some patients with biopsy-confirmed malignancy-associated Sweet's syndrome, fever may be absent. Other Sweet's syndrome-associated symptoms, such as arthralgia, general malaise, headache, and myalgia may also be present [1,2,23].

### Skin lesions

**Figure 4:**
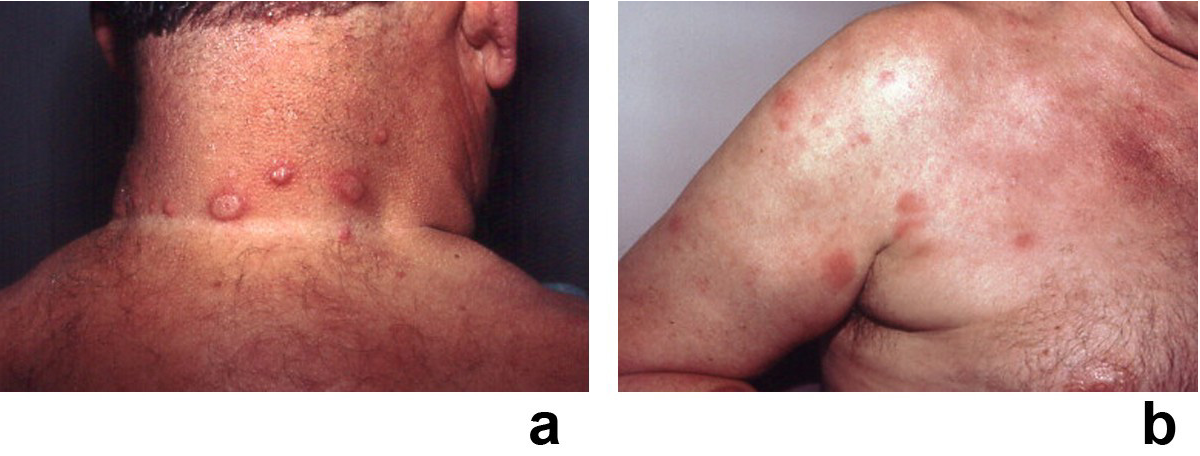
**(a and b)**. Recurrent Sweet's syndrome in a man with antiphospholipid syndrome. A 65-year-old white man with a 7-year history of recurrent Sweet's syndrome and antiphospholipid syndrome (initially manifested by multiple pulmonary emboli and subsequently managed with chronic coumadin therapy). Painful, red, biopsy-confirmed, pseudovesicular nodules and plaques of Sweet's syndrome are present on the posterior neck (a), chest, and upper arm (b). Although the Sweet's syndrome skin lesions resolved after the initiation of oral prednisone (60 mg/day), they recurred when the dose of the drug was subsequently tapered; potassium iodide, colchicine, dapsone, antimalarials, azathioprine, methotrexate, retinoids, and nonsteroidal anti-inflammatory agents were not effective as corticosteroid-sparing agents. (From **[1] **Cohen PR, Kurzrock R: Sweet's syndrome revisited: a review of disease concepts. Int J Dermatol 2003;42:761–778. *Copyright 2003, Reprinted with permission from the International Society of Dermatology, Blackwell Publishing, Oxford, United Kingdom*.)

Skin lesions of Sweet's syndrome are typically tender. They appear as painful, red or purple-red, papules or nodules. Larger lesions may develop into plaques (Figures 1 and 2). The eruption is often distributed asymetrically. It presents as either a single lesion or multiple lesions. The most frequent lesion locations are the upper extremities, face, and neck (Figure 4) [1,10].

The Sweet's syndrome lesions have a transparent, vesicle-like appearance because of the pronounced edema in the upper dermis; some lesions are morphologically similar to bullae [401,419]. Central clearing may lead to annular or arcuate patterns in latter stages. In patients with malignancy-associated Sweet's syndrome, the lesions may appear bullous, become ulcerated, and/or mimic the morphologic features of pyoderma gangrenosum [133,134,401].

The individual Sweet's syndrome lesions enlarge and may coalesce to form irregular, sharply border plaques over a period of days to weeks. Subsequently, either spontaneously or after treatment, the lesions usually resolve without scarring. In one-third to two-thirds of patients, lesions associated with recurrent episodes of Sweet's syndrome occur [1,2,135,136].

**Figure 5:**
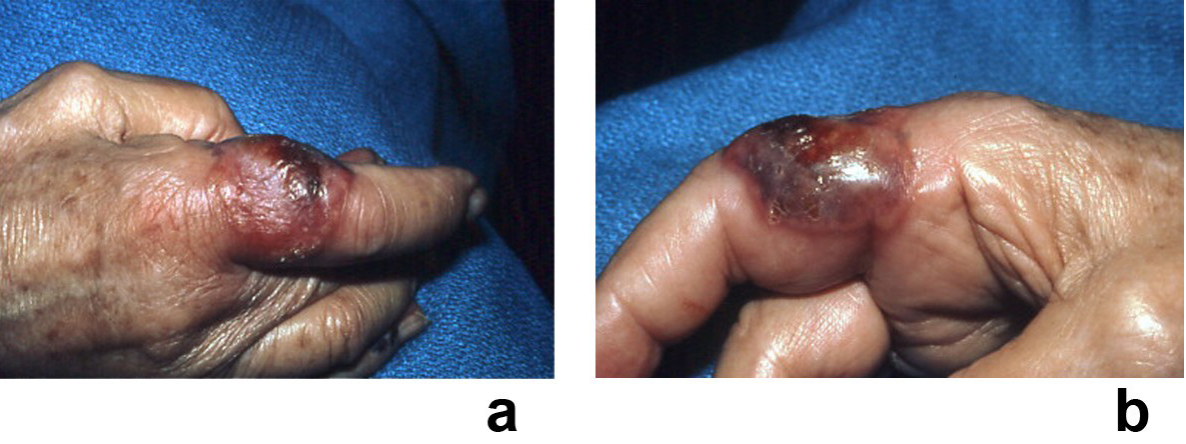
**(a and b)**. Radiotherapy-related Sweet's syndrome skin lesions in a woman with malignancy-associated Sweet's syndrome. Radiotherapy associated-exacerbated Sweet's syndrome in a patient with chronic lymphocytic leukemia and cutaneous squamous cell carcinoma. This biopsy-confirmed culture-negative, erythematous-based hemorrhagic, and vesicular-appearing Sweet's syndrome lesion extends from the dorsal (a) to the palmar (b) surface of the radial side of the right index finger and involves the skin between the metacarpal-phalangeal joint and the proximal interphalangeal joint of a 77-year-old woman. She has a prior medical history of hypothyroidism for which she receives daily Synthroid. Twenty-four months earlier, she had been diagnosed with chronic lymphocytic leukemia, which is adequately being managed with 2 mg of Myleran each day; her current white blood cell count of 51,800 cells/mm^3 ^consists of 79% neutrophils, 15% bands, and 2% lymphocytes. More recently (3 months previously), a biopsy confirmed (at both initial microscopic evaluation and subsequent consultation pathology review) cutaneous squamous cell carcinoma involving the left index finger. She was started on an oral antibiotic (ciprofloxaxin), and radiotherapy (in fractionated doses over a period of 3 weeks) to the left index finger tumor was performed; during this treatment, a clinically similar-appearing lesion whose "presentation was suspicious for squamous cell carcinoma" began to develop on her right index finger. Based only on the lesion's morphologic characteristics, a single treatment with radiotherapy was also given; promptly thereafter, the right index finger lesion rapidly increased in size. Within a week, the lesion on the right index finger (as shown in a and b) had grown to a 4- × 4-cm pseudovesicular nodule that nearly involved the entire circumference of the digit and she was referred to the dermatology clinic. The lesion was painful and her leukocyte count was markedly elevated; however, she was (and had been) afebrile. Lesional biopsies for microscopic and culture evaluation were performed. Because the diagnosis of Sweet's syndrome was suspected, daily oral corticosteroid therapy with 60 mg prednisone was started. Oral cephalexin (250 mg 4 times each day for 7 days) and topical 2% mupirocin ointment (3 times each day) were also given. After a week of therapy, the lesion was greatly improved: it was no longer tender and it had decreased in size. Subsequently, the lesion completely resolved. The dose of prednisone was tapered during the next 5 weeks and then discontinued. (From **[23] **Cohen PR, Kurzrock R: Sweet's syndrome: a neutrophilic dermatosis classically associated with acute onset and fever. Clin Dermatol 2000;18:265–282. *Copyright 2000, Reprinted with permission from Elsevier Ltd, Oxford, United Kingdom*.)

Skin hypersensitivity, also referred to as cutaneous pathergy, is a Sweet's syndrome-associated feature characterized by dermatosis-associated skin lesions appearing at sites of cutaneous trauma [1,2,410,432]. These include the sites where procedures such as biopsies [20], intravenous catheter placement [20,400], vaccination [419], and venipuncture have been performed [12,17,20,37,137,138]. Sweet's syndrome lesions have also been observed at the locations of cat scratches and insect bites [20], areas that have received radiation therapy (Figure 5) [23,139-141], and places that have been contacted by sensitizing antigens [137,142]. Lesions have also been photodistributed (Figures 3a and 3b) or localized to the site of a prior phototoxic reaction (sunburn) in some Sweet's syndrome patients [13,20,98,143-145]. Occasionally, Sweet's syndrome lesions have appeared on the arm affected by postmastectomy lymphedema [100,146,414].

In addition to pseudovesicular papules, plaques, and nodules, Sweet's syndrome can appear as a pustular dermatosis [147]. The lesions appear as either erythematous-based pustules or tiny pustules on the tops of red papules. This clinical variant of Sweet's syndrome probably also includes the "pustular eruption of ulcerative colitis" that has previously been described in some of the patients with this inflammatory bowel disease [1,148].

When the clinical lesions of the dermatosis are predominantly restricted to the dorsal hands, this localized, pustular variant of Sweet's syndrome has been referred to as either "neutrophilic dermatosis of the dorsal hands" or "pustular vasculitis of the dorsal hands" [3,149-154,401,402,408,420,427]. The morphology and response to treatment of the dermatosis-associated lesions reported as neutrophilic dermatosis of the dorsal hands are similar to those of Sweet's syndrome. The lesions rapidly resolve after systemic corticosteroids and/or dapsone therapy is initiated. Resolution of lesions has also been observed spontaneously [420] or following therapy with either topical corticosteroids [401], systemic colchicine [401], or systemic indomethacin [427]. In addition, concurrent lesions were located on either their oral mucosa, arm, leg, back, and/or face of several patients with this form of the disease [3,155-163,401,420].

**Figure 6:**
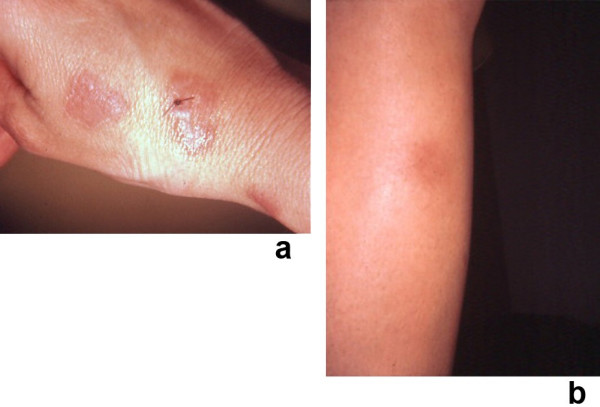
**(a and b)**. Clinical presentation of concurrent Sweet's syndrome and erythema nodosum. A 30-year-old woman had been receiving an oral contraceptive (0.3 mg norgestrel with 0.3 mg ethinyl estradiol) and an appetite suppressant (105 mg phendimetrazine tartrate) during the 5 months before these dermatoses appeared. An episode of recurrent herpes labialis involving her right upper lip also began 2 days prior to the onset of her skin lesions. Tender, erythematous plaques of biopsy-confirmed Sweet's syndrome are shown on the dorsal right hand and wrist; the suture indicates the biopsy site (a). A tender, erythematous nodule of biopsy confirmed erythema nodosum – which morphologically mimiced subcutaneous Sweet's syndrome – is shown on her right pretibial region (b). (From **[21] **Cohen PR, Holder WR, Rapini RP: Concurrent Sweet's syndrome and erythema nodosum: a report, world literature review and mechanism of pathogenesis. J Rheumatol 1992;5:814–820. *Copyright 1992, Reprinted with permission from the Journal of Rheumatology, Toronto, Ontario*.)

**Figure 7:**
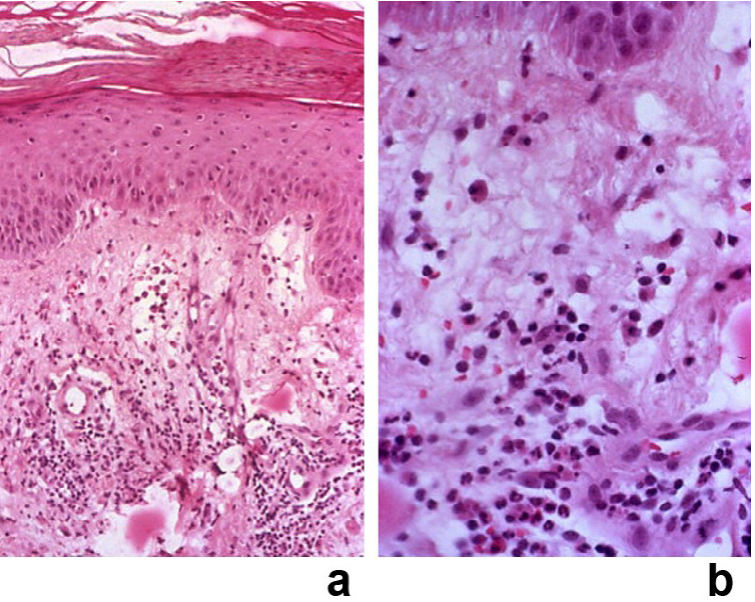
**(a and b)**. Histopathology of a Sweet's syndrome lesion from a patient with concurrent erythema nodosum. Skin biopsy from one of the Sweet's syndrome plaques on the right dorsal wrist of a woman with concurrent biopsy-confirmed Sweet's sydrome and biopsy-confirmed erythema nodosum. The characteristic histopathologic features of Sweet's syndrome are observed at low (a) and higher (b) magnification: papillary dermal edema, swollen endothelial cells, and a diffuse infiltrate of predominantly neutrophils with leukocytoclasia. There is no evidence of vasculitis (hematoxylin and eosin, × 25 (a), × 100 (b)). (From **[21] **Cohen PR, Holder WR, Rapini RP: Concurrent Sweet's syndrome and erythema nodosum: a report, world literature review and mechanism of pathogenesis. J Rheumatol 1992;5:814–820. *Copyright 1992, Reprinted with permission from the Journal of Rheumatology, Toronto, Ontario*.)

Subcutaneous Sweet's syndrome is characterized by skin lesions which usually present as erythematous, tender dermal nodules on the extremities [4,8,12,17,99,119,164-185,397]. The lesions often mimic erythema nodosum when they are located on the legs [170]. Even in a patient whose Sweet's syndrome has previously been biopsy-confirmed, tissue evaluation of one or more new dermal nodules may be necessary to establish the correct diagnosis since Sweet's syndrome can present concurrently (Figures 6 and 7) [21,125,187-189] or sequentially [170] with erythema nodosum [1,2,4,17,21,187,190,403].

### Extracutaneous manifestations

Bones, central nervous system, ears, eyes, kidneys, intestines, liver, heart, lung, mouth, muscles, and spleen can be the sites of extracutaneous manifestations of Sweet's syndrome (Table 4) [12,16,17,20,25,26,32,33,44,73,75,88,101,117,138,139,165,202,203,205,212-257,421,423,434]. In addition, dermatosis-related sterile osteomyelitis has been reported in children.

**Table 4:** Extracutaneous manifestations of Sweet's syndrome

Bone	Acute sterile arthritis, arthralgias, focal aseptic osteitis, pigmented villonodular synovitis, sterile osteomyelitis (chronic recurrent multifocal osteomyelitis) [12,32,44,164,212-215].
Central nervous system	Acute benign encephalitis, aseptic meningitis, brain SPECT abnormalities, brain stem lesions, cerebrospinal fluid abnormalities, computerized axial tomography abnormalities, electroencephalogram abnormalities, encephalitis, Guillain-Barre syndrome, idiopathic hypertrophic cranial pachymeningitis, idiopathic progressive bilateral sensorineural hearing loss, magnetic resonance imaging abnormalities, neurologic symptoms, "neuro-Sweet disease", pareses of central origin, polyneuropathy, psychiatric symptoms [33,68,212-229,423].
Ears	Tender red nodules and pustules that coalesced to form plaques in the external auditory canal and the tympanic membrane [230].
Eyes	Blepharitis, conjunctival erythematous lesions with tissue biopsy showing neutrophilic inflammation, conjunctival hemorrhage, conjunctivitis, dacryoadenitis, episcleritis, glaucoma, iridocyclitis, iritis, limbal nodules, ocular congestion, periocular swelling, peripheral ulcerative keratitis, retinal vasculitis, scleritis, uveitis [12,20,26,88,101,185,202,214,229,231-239,421].
Kidneys	Mesangiocapillary glomerulonephritis, urinalysis abnormalities (hematuria and proteinuria) [16,17,25,26,73].
Intestines	Intestine with extensive and diffuse neutrophilic inflammation, neutrophilic ileal infiltrate, pancolitis (culture-negative) [36,203,240,241].
Liver	Hepatic portal triad with neutrophilic inflammation, hepatic serum enzyme abnormalities, hepatomegaly [12,16,17,20,25,26,212,224,242].
Heart	Aortic stenosis (segmental), aortitis (neutrophilic and segmental), cardiomegaly, coronary artery occlusion, heart failure, myocardial infiltration by neutrophils, vascular (aorta, bracheocephalic trunk and coronary arteries) dilatation [243-247].
Lung	Bronchi (main stem) with red-bordered pustules, bronchi with neutrophilic inflammation, pleural effusion showing abundant neutrophils without microorganisms, progressive pharyngeal mucosal infiltration and edema resulting in upper-airway obstruction, and chest roentgenogram abnormalities: corticosteroid-responsive culture-negative infiltratives, pulmonary tissue with neutrophilic inflammation [17,20,73,101,138,139,165,205,212,246-251,434].
Mouth	Aphthous-like superficial lesions (buccal mucosa, tongue), bullae and vesicles (hemorrhagic: labial and gingival mucosa), gingival hyperplasia, necrotizing ulcerative periodontitis, nodules (necrotic: labial mucosa), papules (macerated: palate and tongue), pustules (individual and grouped: palate and pharynx), swelling (tongue), ulcers (buccal mucosa and palate) [26,75,102,117,203,249,252-254].
Muscles	Magnetic resonance imaging (T1-weighted and T2-weighted) abnormalities: high signal intensities due to myositis and fasciitis, myalgias (in up to half of the patients with idiopathic Sweet's syndrome), myositis (neutrophilic), tendinitis, tenosynovitis [73,75,244,255-257].
Spleen	Splenomegaly [212].

Source **[1]**: Adapted with permission from Cohen PR, Kurzrock R: Sweet's syndrome revisited: a review of disease concepts. Int J Dermatol 2003;42:761–778. *Copyright 2003, Reprinted with permission from the International Society of Dermatology, Blackwell Publishing Ltd, Oxford, United Kingdom*.

Ocular manifestations may be the presenting feature of Sweet's syndrome. The incidence of ocular involvement (such as conjunctivitis) is variable in classical Sweet's syndrome. However, ocular lesions of Sweet's syndrome are uncommon in the malignancy-associated and drug-induced forms of the dermatosis.

Mucosal involvement of the mouth, appearing as oral ulcers, is uncommon in patients with classical Sweet's syndrome. However, dermatosis-related oral lesions occurs more frequently in Sweet's syndrome patients with hematologic disorders [23,26,102,117,203,252]. Similar to extracutaneous manifestations of Sweet's syndrome occurring at other sites, the dermatosis-related oral ulcers typically resolve after initiation of treatment with systemic corticosteroids [1,2].

#### Included diseases

Several conditions – including other neutrophilic dermatoses and leukemia cutis – have been observed to occur either before, concurrent with, or following the diagnosis of Sweet's syndrome in patients with this dermatosis. Therefore, it is reasonable to conclude that the occurrence of Sweet's syndrome in these individuals may be etiologically to the development of some of these conditions.

##### Associated diseases

**Figure 8:**
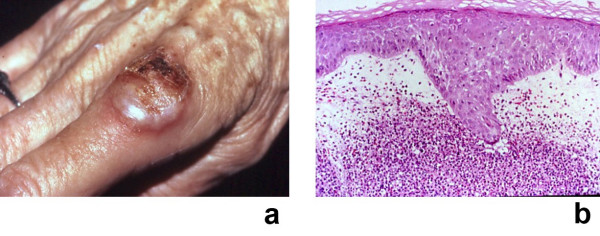
**(a and b)**. Malignancy-associated Sweet's syndrome in a woman with dermatosis-related laryngeal carcinoma. Solid tumor-associated Sweet's syndrome in a 69-year-old woman in whom a workup after the appearance of Sweet's syndrome lesions revealed an unsuspected recurrent squamous cell carcinoma of the larynx. A tender, erythematous-based, vesicular-appearing nodule of Sweet's syndrome with central ulceration and crust located proximally on the dorsum of the right second digit is shown (a). The histologic features of the Sweet's syndrome lesion revealed a neutrophilic dermatosis; the papillary dermis was edematous and contained a dense infiltrate of neutrophils (b) (hematoxylin and eosin, × 50). (From **[362] **Cohen PR, Holder WR, Tucker SB, Kono S, Kurzrock R: Sweet's syndrome in patients with solid tumors. Cancer 1993;72:2723–2731. *Copyright 1993, Reprinted with permission from John Wiley & Sons, Inc., Hoboken, New Jersey*.)

**Figure 9:**
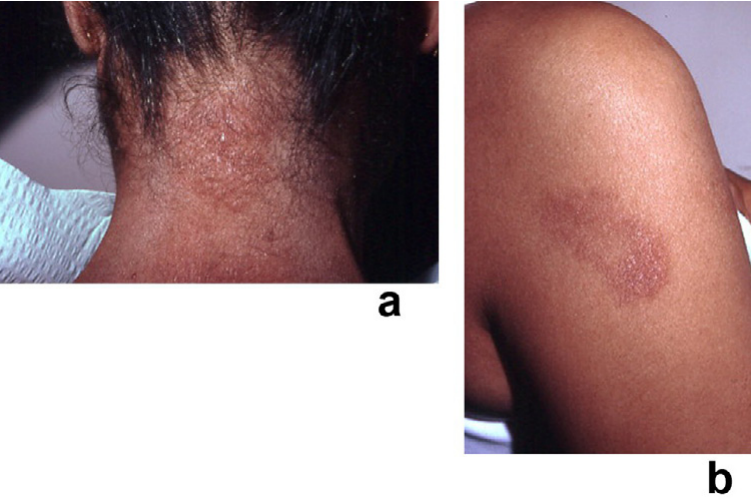
**(a and b)**. Recurrent, oral corticosteroid-responsive, Sweet's syndrome lesions. A 46-year-old Indian woman with oral corticosteroid-responsive, tender erythematous, biopsy-confirmed plaques of recurrent Sweet's syndrome on the posterior neck (a) and right arm (b). Her laboratory evaluation was significant for the elevation of the erythrocyte sedimentation rate, liver function tests [serum glutamic-oxaloacetic transaminase (SGOT), serum glutamic-pyruvic transaminase (SGPT), and lactate dehydrogenase (LDH)], cytomegalovirus immunoglobulin G (IgG) antibody (consistent with previous exposure), hepatitis A IgG antibody (consistent with previous infection), and Epstein-Barr virus capsid IgG antibody (consistent with recent or past infection). (From **[1] **Cohen PR, Kurzrock R: Sweet's syndrome revisited: a review of disease concepts. Int J Dermatol 2003;42:761–778. *Copyright 2003, Reprinted with permission from the International Society of Dermatology, Blackwell Publishing, Oxford, United Kingdom*.)

A *bona fide *association between Sweet's syndrome probably exists with the following conditions: cancer (including both hematologic malignancies and solid tumors) (Figure 8), infections (predominantly of the upper respiratory tract and the gastrointestinal tract), inflammatory bowel disease (including both Crohn's disease and ulcerative colitis), medications (granulocyte-colony stimulating factor is the most commonly reported drug) and pregnancy (Table 5). There are also several conditions for which the association with Sweet's syndrome is possibly bona fide (Table 6). In addition, the validity of the association between Sweet's syndrome and many of the conditions that have been observed in patients with the dermatosis remains to be established (Table 7) (Figure 9); indeed, the detection of that condition in an individual with Sweet's syndrome may merely represent a coincidental occurrence [1,2,5,11-20,30,36-43,69-126,158-161,164,166,186-190,195,214,231,236,259-339,401,404,407,409-411,413,415,417-419,421,424,425,431,433].

**Table 5:** Sweet's syndrome and probably associated conditions

Cancer	Hematologic malignancies (most commonly acute myelogenous leukemia) and solid tumors (most commonly carcinomas of the genitourinary organs, breast, and gastrointestinal tract) [15]
Infections [12,16,17,19,20,125,126,166,186,259]	Most commonly of the upper respiratory tract (streptococcosis) [16,17,20,186] and the gastrointestinal tract (salmonellosis [19,166] and yersiniosis [12])
Inflammatory bowel disease [260]	Crohn's disease [12,16,17,20,30,115,164,187,261-267,411] and ulcerative colitis [12,16-18,20,30,214,268,269]
Medications	Most commonly granulocyte colony-stimulating factor [11,13,17,39,41,69-124,404,417]
Pregnancy [12,16,30,270,271,410]	

Source **[1]**: Adapted with permission from Cohen PR, Kurzrock R: Sweet's syndrome revisited: a review of disease concepts. Int J Dermatol 2003;42:761–778. *Copyright 2003, Reprinted with permission from the International Society of Dermatology, Blackwell Publishing Ltd, Oxford, United Kingdom*.

**Table 6:** Sweet's syndrome and possibly associated conditions

Behcet's disease [272]
Erythema nodosum [17,30,186,187,190,214,236,264,266,273-276,405,415]
Relapsing polychondritis [5,20,195,277-280,409,425]
Rheumatoid arthritis [12,16,20,231]
Sarcoidosis [18,188,274,281-283,409,413]
Thyroid disease: Grave's disease [117,284,285] and Hashimoto's thyroiditis [12,286]

Source **[1]**: Adapted with permission from Cohen PR, Kurzrock R: Sweet's syndrome revisited: a review of disease concepts. Int J Dermatol 2003;42:761–778. *Copyright 2003, Reprinted with permission from the International Society of Dermatology, Blackwell Publishing Ltd, Oxford, United Kingdom*.

**Table 7:** Conditions for which the validity of their association in Sweet's syndrome patients remains to be established

Alpha 1-antitrypsin deficiency [42]
Anti-factor VIII inhibitor [287]
Antiphospholipid syndrome [1]
Aortitis (Takayasu's arteritis) [286,288]
Aplastic anemia [39,99]
Autoimmune disorders: autoimmune thrombocytopenic purpura [401], connective tissue disease (undifferentiated) [401], dermatomyositis [20], lupus erythematosus (subacute [289] and systemic [119,290,401]), pemphigus vulgaris [291] and Sjogren's syndrome [12,17,265,433]
Bronchiolitis obliterans and organizing pneumonia [292-294]
Chemical fertilizer [158]
Chronic fatigue syndrome [295]
Cirrhosis (cryptogenic) [30]
Cholelithiasis [421]
Common bile duct and intrahepatic duct stones [19]
Congenital dyserythropoietic anemia [18]
Congenital neutropenia (Kostmann's syndrome) [97]
Cutis laxa (acquired, Marshall syndrome) [38,42,245,246]
Dressler's syndrome (postmyocardial infarction syndrome) [17]
Eosinophilic granuloma [19]
Fanconi anemia [36,241]
Glycogen storage disease (Type Ib) [41]
Granuloma annulare [296]
IgA nephropathy (Berger's disease) [97]
Immunizing agent (BCG vaccination and flu) [17,297,298,419]
Immunodeficiency diseases: chronic granulomatous disease [37,299,300], complement deficiency [30], human immunodeficiency virus infection [98,254,302], and primary T-cell immunodeficiency disease [43,303])
Infections: *Anaplasma phagocytophilum *[304], bartholinitis [17], bronchitis [17], *Capnocytophaga canimorsus *[305], chlamydia [306-308], cholangitis [19], cholecystitis [12], coccidioidomycosis [309], cytomegalovirus [1,310,311], *Entamoeba histolytica *[214], Epstein-Barr virus [1], *Francisella tularensis *[312], *Helicobacter pylori *[313], hepatitis (acute hepatitis B [314], autoimmune [251], cholestatic [30], chronic active [315], hepatitis C [161], and prior hepatitis A [1]), herpes simplex [316,317], herpes zoster [119], histoplasmosis [259], human immunodeficiency virus [98,254,302], leprosy [19], lymphadenitis (not otherwise specified [318,319] and subacute necrotizing [212,318]), mycobacteria (nontuberculous) [19,212,316,320-322,401], otitis media [12,17], pancreatitis [30], *Pasteurella multocida *bronchitis [323], *Penicillium *species [401], *Pneumocystis carinii *pneumonia [324], pyelonephritis [12], *Salmonella *(group D cervical lymphadenitis) [401], *Staphylococcus aureus *[44,326,431], *Staphylococcus epidermidis *(methicillin resistant) [407], subacute bacterial endocarditis [327], tonsillitis [12,17,19], toxoplasmosis [328], *Trichophyton rubrum *[418], tuberculosis [17,329,330], ureaplasmosis [331], urinary tract [17,20], and vulvovaginitis [12,20]
Malabsorption [30]
Mid-dermal elastolysis [332]
POEMS syndrome (polyneuropathy, organomegaly, endocrinopathy, M protein, and skin changes) [333]
Postoperative (pneumonectomy) [334]
Psoriasis vulgaris [16]
Rhinosinusitis [335]
SAPHO syndrome (synovitis, acne, pustulosis, hyperostosis, and osteomyelitis) [424]
Still's disease [336]
Thermal injury [160]
Transient acantholytic dermatosis (Grover's disease) [337]
Ureter obstruction [338]
Urticaria (chronic) [17]
Urticaria pigmentosa [12]
Welding burns [339]

Source **[1]**: Adapted with permission from Cohen PR, Kurzrock R: Sweet's syndrome revisited: a review of disease concepts. Int J Dermatol 2003;42:761–778. *Copyright 2003, Reprinted with permission from the International Society of Dermatology, Blackwell Publishing Ltd, Oxford, United Kingdom*.

##### Associated neutrophilic dermatoses

Neutrophilic dermatoses of the skin and mucosa demonstrate a unifying characteristic. They all have an inflammatory infiltrate that consists of mature polymorphonuclear leukocytes. Although many of these conditions exhibit similar clinical and pathologic features, the location of the neutrophilic infiltrate may permit differentiation of these dermatoses [6,120,416]. Erythema elevatum diutinum [340], neutrophilic eccrine hidradenitis [6], pyoderma gangrenosum [231,269,341,342,401], subcorneal pustular dermatosis [6], and vasculitis [3,192,210,231] are other neutrophilic dermatoses that have been observed to occur in patients with Sweet's syndrome. The onset of Sweet's syndrome may appear prior to, concurrent with, or following the detection of the additional neutrophilic dermatoses.

##### Associated leukemia cutis

Sweet's syndrome may occur in three settings in patients with hematologic disorders, such as leukemia: (1) a paraneoplastic syndrome heralding either the initial discovery of an unsuspected malignancy or the recurrence of a previously treated cancer; (2) a drug-induced dermatosis subsequent to the patient being treated with either all-trans retinoic acid, bortezomib, granulocyte-colony stimulating factor, or imatinib mesylate; or (3) a dermatosis in which the skin lesions concurrently demonstrate leukemia cutis [1]. In the latter setting, the individual skin lesions are characterized by the coincident presence of abnormal neutrophils (leukemia cutis) and mature polymorphonuclear leukocytes (Sweet's syndrome) [1,70,71,93,109,165,205-211,401,417,425]. The most frequent hematologic malignancies associated with this unique presentation of Sweet's syndrome are acute myelocytic leukemia and acute promyelocytic leukemia. Other hematologic disorders that have been associated with concurrent Sweet's syndrome and leukemia cutis are chronic myelogenous leukemia, myelogenous leukemia (not otherwise specified), and myelodysplastic syndrome [109].

Several hypotheses have been suggested to explain concurrent Sweet's syndrome and leukemia cutis in the same lesion. One theory is that the circulating immature myeloid precursor cells are innocent bystanders that have been recruited to the skin as the result of an inflammatory oncotactic phenomenon stimulated by the Sweet's syndrome lesions ("secondary" leukemia cutis) [165,206,207]. Alternatively, the leukemic cells within the skin may constitute the bonified incipient presence of a specific leukemic infiltrate ("primary" leukemia cutis) [207]. Finally, in patients with "primary" leukemia cutis who have been treated with this granulocyte-colony stimulating factor, it is possible that the atypical cells of leukemia cutis developed into mature neutrophils of Sweet's syndrome as a result of granulocyte-colony stimulating factor therapy-induced differentiation of the sequestered leukemia cells [205].

## Pathological description

The diagnostic criteria for Sweet's syndrome includes a diffuse infiltrate of mature neutrophils. In addition to the dense polymorphonuclear cell infiltrate in the upper dermis, edema are characteristically present (Figures 8b and 3c). Fragmented neutrophil nuclei (referred to as karyorrhexis or leukocytoclasia), swollen endothelial cells, and dilated small blood vessels may also be present (Figure 7). The overlying epidermis is normal and changes of "primary" leukocytoclastic vasculitis (such as fibrin deposition or neutrophils within the vessel walls) are usually absent [1,2,23,167,168].

Since the initial description of 'acute febrile neutrophilic dermatosis' by Dr. Sweet, the spectrum of pathologic changes described in cutaneous lesions of Sweet's syndrome has expanded. There is variability regarding the composition of the inflammatory infiltrate and its depth within the skin. Although the neutrophilic infiltrate is traditionally found in the dermis, it can also be present in either the overlying epidermis or the underlying adipose tissue. Also, in patients with hematologic malignancy-associated Sweet's syndrome, concurrent leukemia cutis may be present in the dermatosis-related skin lesions [3,191].

The predominant cells that comprise the infiltrate in the dermis of cutaneous Sweet's syndrome lesions are mature neutrophils. However, eosinophils have been observed within the dermal infiltrate in the Sweet's syndrome skin lesions of some patients with either the classical [11,167,168,195,202-204,212] or the drug-induced [84,107,110,111] dermatosis. Occasionally, lymphocytes or histiocytes may also be present in the inflammatory infiltrate [11,104,167,168,198-200,425].

A unique clinicopathologic subset of 11 Sweet's syndrome patients has recently been characterized [200,425]. The patients either presented with or subsequently developed myelodysplastic syndrome; four of the patients also developed relapsing polychondritis [5,409,425]. Initially, the diagnosis of Sweet's syndrome was based on clinical features. Surprisingly, the Sweet's syndrome lesions from the early episodes of the dermatosis in these individuals showed a dense mononuclear cell infiltrate consisting predominantly of lymphocytes; histiocytes and atypical mononuclear cells were also present. However, 24 to 96 months later, sequential biopsies of lesion from recurrent episodes in these patients revealed neutrophilic dermal infiltrates typical of Sweet's syndrome [425].

Abnormal or immature myeloid cells have been observed in Sweet's syndrome lesions. For example, abnormal neutrophils (leukemia cutis) – in addition to mature neutrophils – comprise the dermal infiltrate in some Sweet's syndrome patients with hematologic disorders [1,70,71,93,109,165,205-211,401,417]. Recently, a "histiocytoid" pathologic variant of Sweet's syndrome has been described; it is characterized by an infiltrate mainly composed of immature myeloid cells which have been misinterpreted as histiocytes (macrophages) [201].

The inflammatory infiltrate in Sweet's syndrome is usually located in the papillary and upper reticular dermis. However, neutrophils can also be present in the epidermis [17,197] or adipose tissue. For example, exocytosis of neutrophils into the overlying epidermis has been observed as either neutrophilic spongiotic vesicles [194] or subcorneal pustules [12,80,167,195,196]. The condition is referred to as "subcutaneous Sweet's syndrome" when the neutrophils are located either entirely or only partially in the subcutaneous fat [4,8,12,17,99,119,164-184,397,399,417].

Subcutaneous Sweet's syndrome can involve either the adipose tissue alone or both the dermis and the subcutaneous fat [1,4,401]. Within the subcutaneous fat, the neutrophilic infiltrate is present in the lobules 166,167,171-173,399,401,417], the septae [167,397], or both [167,174-177]. The presence of subcutaneous neutrophilic inflammation in Sweet's syndrome lesions may be a more common finding in patients with either an associated hematologic dyscrasia [99,165,170,174,177,178,183,184,401,417] or solid tumor [397,399].

Cutaneous lesions of subcutaneous Sweet's syndrome typically present as tender erythematous subepidermal nodules on the extremities. They are morphologically similar in appearance to erythema nodosum [431]. Therefore, a biopsy of one or more new nodules may be necessary to establish the correct diagnosis – even in a patient with histology – confirmed Sweet's syndrome – since Sweet's syndrome can develop concurrently or sequentially with erythema nodosum [21,405].

The neutrophils are typically located in the papillary and upper reticular dermis as a dense and diffusely distributed infiltrate in Sweet's syndrome lesions. However, in some Sweet's syndrome lesions, the neutrophils have been observed to be perivascular and exhibiting pathologic changes consistent with leukocytoclastic vasculitis [192,193,396,401,410,428]. In these lesions of Sweet's syndrome, the vascular changes are considered to be those of a "secondary" leukocytoclastic vasculitis occurring as an epiphenomenon and not representative of a "primary" vasculitis [3]. However, some authors have introduced an alternative hypothesis: that Sweet's syndrome be regarded as a variant of leukocytoclastic vasculitis [428].

Pathologic findings of Sweet's syndrome can also occur in extracutaneous sites. They often present as sterile neutrophilic inflammation in the involved organ. These changes have been described in the bones, intestines, liver, aorta, lungs, and muscles of patients with Sweet's syndrome.

## Etiology

The pathogenesis of Sweet's syndrome remains to be definitively determined. Indeed, it may be multifactorial and many etiologies – not necessarily mutually exclusive – have been postulated. The accompanying fever and peripheral leukocytosis suggest a septic process. Since most patients with classic Sweet's syndrome have a febrile upper respiratory tract infection or tonsillitis that precedes their skin lesions by 1 to 3 weeks, a bacterial infection may have a causative role. Also, the manifestations of Sweet's syndrome improve with systemic antibiotics in some of the patients with dermatosis-associated culture-confirmed and serology-confirmed *Yersinia enterolitica *intestinal infection [2,77,125-127].

A hypersensitivity reaction to an eliciting bacterial, viral, or tumor antigen may promote the development of Sweet's syndrome. This concept is suggested by the appearance, histopathology and course of the Sweet's syndrome skin lesions. This hypothesis is also supported by the prompt response of both the symptoms and the lesions to corticosteroids [2,127].

Circulating autoantibodies, cytokines, dermal dendrocytes, human leukocyte antigen serotypes, immune complexes, and leukotactic mechanisms have all been postulated to contribute to the pathogenesis of Sweet's syndrome. However, complement does not appear to be essential to the disease process. Antibodies to neutrophilic cytoplasmic antigens have been demonstrated in some Sweet's syndrome patients. Yet, their role in the pathogenesis of this dermatosis has not been established and they are likely to represent an epiphenomenon [2].

The effects of cytokines – either directly or indirectly or both – have an etiologic role in the development of Sweet's syndrome symptoms and lesions [2,21-23]. Granulocyte-colony stimulating factor, granulocyte macrophage colony stimulating factor, interferon-gamma, interleukin-1, interleukin-3, interleukin-6, and interleukin-8 are potential cytokine candidates [2,13,21,23,44,128-132,397,398]. Recently, a healthy woman donor for peripheral blood stem cell harvest was observed to develop granulocyte-colony stimulating factor-induced Sweet's syndrome which appeared four days after commencing granulocyte-colony stimulating factor at a dose of 10 micrograms per kilogram per day [398].

Tumor-associated production of granulocyte-colony stimulating factor may be involved in the development of Sweet's syndrome in patients with dermatosis-related malignancies. Production of granulocyte-colony stimulating factor and marked leukocytosis have been demonstrated by various malignant tumors. Immunohistiochemical studies showed staining for granulocyte-colony stimulating factor in the tumor cells and surrounding matrix of an intrahepatic cholangiocarcinoma which had been resected from a woman whose Sweet's syndrome was associated with this cancer. Although the level of serum granuocyte-colony stimulating factor was not abnormally elevated, the detection of granulocyte-colony stimulating factor in the tumor suggests the possiblity that tumors capable of producing granulocyte-colony stimulating factor might cause Sweet's syndrome in these oncology patients [397].

Another example supporting the role of cytokines in the pathogenesis of Sweet's syndrome is a patient with myelodysplastic syndrome-associated (non-granulocyte-colony stimulating factor-induced) Sweet's syndrome in whom elevated serum levels of granulocyte-colony stimulating factor and interleukin-6 were detected [128]. Also, in an infant with classical Sweet's syndrome, detectable levels of intra-articular synovial fluid granulocyte macrophage-colony stimulating factor were observed [44]. A recent study comparing patients with active and inactive Sweet's syndrome demonstrated significantly higher levels of serum granulocyte-colony stimulating factor in individuals whose dermatosis was active than in patients whose Sweet's syndrome was inactive [129].

Immunohistochemical evaluation of the epidermis of Sweet's syndrome lesions suggests the importance of interleukins as a potential cytokine mediator in Sweet's syndrome. Significantly elevated levels of helper T-cell type 1 cytokines (interleukin-2 and interferon-gamma) and normal levels of a helper T-cell type 2 cytokine (interleukin-4) were observed in the immunohistochemical studies of Sweet's syndrome patients' serum [130]. Decreased epidermal staining for interleukin-1 and interleukin-6 were noted in other immunohistochemical studies; the investigators postulated that these findings were due to the release of these cytokines into the dermis [131]. In summary, granulocyte-colony stimulating factor, granulocyte macrophage colony stimulating factor, interferon-gamma, interleukin-1, interleukin-3, interleukin-6, and interleukin-8 are potential cytokine candidates in the pathogenesis of Sweet's syndrome [2,13,21-23,44,128-132].

The possibility that Sweet's syndrome, when associated with neutrophil monoclonality, represents a form of indolent, localized cutaneous neutrophilic dyscrasia has recently been postulated. An X-inactivation assay to detect clonal restriction of neutrophils, based on the human androgen receptor (*HUMARA*) gene, was performed on Sweet's syndrome skin biopsy specimens from four patients with acute myelogenous leukemia and two patients without underlying hematologic dyscrasia when the biopsies were obtained. Clonal restriction of the neutrophil infiltrate was found in two of the patients with acute myelogenous leukemia; the other two patients were homozygous for the *HUMARA *gene, precluding analysis. Both control patients had clonal restricted infiltrates within their skin lesions; subsequent investigation revealed unexplained neutropenia with a bone marrow biopsy interpreted as being within normal limits and no further features to suggest a definitive myeloproliferative disorder. These findings demonstrated that clonality of the neutrophilic infiltrate in Sweet's syndrome skin lesions is not exclusively restricted to patients with an established myeloproliferative disease. The significance of the clonal neutrophilic infiltrate in Sweet's syndrome patients without an underlying myeloid dysplasia remains to be determined; however, the investigators speculated that it may have some implications regarding the pathogenesis of sterile neutrophilic infiltrates [429].

## Diagnostic methods

### Lesional skin biopsy

A lesional skin biopsy for routine histopathologic evaluation is a useful procedure to confirm a clinically suspected diagnosis of Sweet's syndrome. Pathologic features of Sweet's syndrome, such as the diffuse inflammatory infiltrate of neutrophils in the dermis, subcutaneous fat, or both can also be observed in cutaneous lesions caused by an infectious agent. Therefore, it may also be prudent to also submit lesional tissue for bacterial, fungal, mycobacterial, and possibly viral cultures [1,2].

### Laboratory evaluation

The most consistent laboratory abnormalties in patients with Sweet's syndrome are peripheral leukocytosis with neutrophilia and an elevated erythrocyte sedimentation rate [23]. However, an elevated white blood cell count is not always observed in all patients with biopsy-confirmed Sweet's syndrome [26]. For example, some of the patients with malignancy-associated Sweet's syndrome may have either anemia, neutropenia, and/or abnormal platelet counts.

Extracutaneous manifestations of Sweet's syndrome may result in other laboratory abnormalities. Abnormalites may be found on brain SPECTs, computerized axial tomography, electroencephalograms, magnetic resonance imaging and cerebrospinal fluid analysis in patients with central nervous system involvement. Urinalysis abnormalities (hematuria and proteinuria) may be observed in patients with dermatosis-related kidney involvement. Hepatic serum enzyme elevation may be present in patients with Sweet's syndrome-associated liver involvment. Pleural effusions and corticosteroid-responsive culture-negative infiltrates may be present on chest roentgenograms in patients with Sweet's syndrome who have extracutaneous manifestations that involve their lungs [2,343].

Laboratory evaluation should include a complete blood cell count with leukocyte differential and platelet count. Evaluation of acute phase reactants (such as the erythrocyte sedimentation rate or C-reactive protein), serum chemistries (evaluating hepatic function and renal function) and a urinalysis should also be performed. It may also be reasonable to perform a serologic evaluation for antistreptolysin-O antibody, rheumatoid factor, and thyroid function since streptococcal infection, rheumatoid arthritis, and thyroid disease have been observed to have either a probably or possible bona fide association with the dermatosis [1,2].

Recommendations for the initial malignancy workup in newly diagnosed Sweet's syndrome patients without a prior cancer were proposed by Cohen and Kurzrock [15] in 1993. Their recommendations were based upon the neoplasms that had concurrently or subsequently been discovered in previously cancer-free Sweet's syndrome patients and the age-related recommendations by the American Cancer Society for the early detection of cancer in asymptomatic persons [406]. They recommended: (1) a detailed medical history; (2) a complete physical examination, including: (a) examination of the thyroid, lymph nodes, oral cavity, and skin; (b) digital rectal examination; (c) breast, ovary, and pelvic examination in women; and (d) prostate and testicle examination in men; (3) laboratory evaluation: (a) carcinoembryonic antigen level; (b) complete blood cell count with leukocyte differential and platelet count; (c) pap test in women; (c) serum chemistries; (d) stool guaiac slide test; (e) urinalysis; and (f) urine culture; and (4) other screening tests: (a) chest roentgenograms; (b) endometrial tissue sampling in either menopausal women or women with a history of abnormal uterine bleeding, estrogen therapy, failure to ovulate, infertility, or obesity; and (c) sigmoidoscopy in patients over 50 years of age. They also suggested that it was reasonable to check a complete blood cell count with leukocyte differential and platelet count every 6 to 12 months since the intial appearance of dermatosis-related skin lesions preceded the diagnosis of a Sweet's syndrome-associated hematologic malignancy by as long as 11 years [2,15].

## Differential diagnosis

### Clinical differential diagnosis

**Figure 10:**
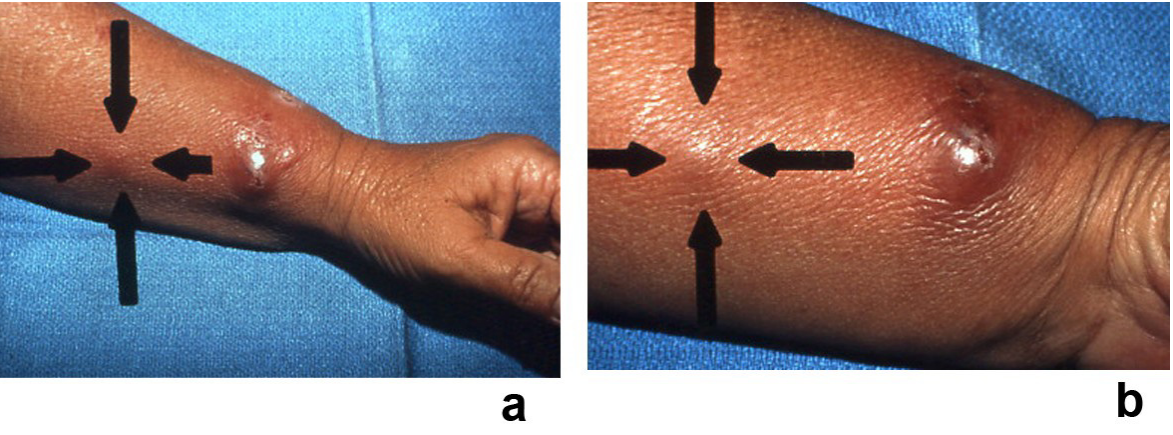
**(a and b)**. Sweet's syndrome with lesions distributed in a sporotrichoid pattern. A 53-year-old Hispanic woman presented to the emergency room with a recent episode of a "sore throat," fever, and a 2-day history of a swollen, tender left wrist accompanied by erythema that was beginning to extend proximally; a bacterial cellulitis was clinically suspected and she was started on oral antibiotics: double-strength trimethoprim-sulfamethoxazole twice daily and 400 mg of ofloxacin twice daily. She was initially seen in the dermatology clinic 9 days later; she was still febrile and her original lesion had developed into a painful larger pseudovesicular nodule on the radial side of her left wrist. In addition, distal (a) and closer (b) views show a smaller red dermal nodule (between arrows) that appeared on her left arm proximal to the original lesion. The clinical differential diagnosis included infections whose lesions demonstrated a sporotrichoid pattern (sporotrichosis and atypical mycobacterial infection) and Sweet's syndrome. Biopsies for microscopic and culture evaluation were performed. In addition to her antibiotics, the patient was started on oral saturated solution of potassium iodide (3 drops 3 times each day and increased by 1 drop each day to a final dose of 10 drops 3 times each day). The hematoxylin and eosin-stained sections from her biopsy showed a neutrophilic dermatosis; the bacterial, mycobacterial, and fungal cultures were negative for organisms. Within a few days after initiating treatment with potassium iodide, her symptoms resolved and her skin lesions began to improve. (From **[23] **Cohen PR, Kurzrock R: Sweet's syndrome: a neutrophilic dermatosis classically associated with acute onset and fever. Clin Dermatol 2000;18:265–282. *Copyright 2000, Reprinted with permission from Elsevier Ltd, Oxford, United Kingdom*.)

There are several mucocutaneous and systemic disorders whose dermatologic manifestations can morpholocally mimic those of Sweet's sydrome. These disorders consist of not only cutaneous conditions and systemic diseases, but also infectious and inflammatory disorders, neoplastic conditions, reactive erythemas, and vasculitis (Figure 10). The clinical differential diagnosis of Sweet's syndrome is listed in Table 8[2,15,23,148,165,194,202,220,344,345,400,412,422].

**Table 8:** Clinical differential diagnosis of Sweet's syndrome

Cutaneous conditions	Acral erythema Drug eruptions Halogenoderma Rosacea fulminans
Infectious and inflammatory disorders	Bacterial sepsis Cellulitis Erysipelas Herpes simplex virus Herpes zoster virus Leprosy Lymphangiitis Panniculitis Pyoderma gangrenosum Syphilis Systemic mycoses Thrombophlebitis Tuberculosis Viral exanthem
Neoplastic conditions	Chloroma Leukemia cutis Lymphoma Metastatic tumor
Reactive erythemas	Erythema multiforme Erythema nodosum Urticaria
Systemic diseases	Behcet's disease Bowel bypass syndrome Dermatomyositis Familial Mediterranean fever Lupus erythematosus
Vasculitis	Erythema elevatum diutinum Granuloma faciale Leukocytoclastic vasculitis Periarteritis nodosa

Source **[15]**: Adapted with permission from Cohen PR, Kurzrock R: Sweet's syndrome and cancer. Clin Dermatol 1993;11:149–157. *Copyright 1993, Reprinted with permission from Elsevier Ltd, Oxford, United Kingdom*.

### Histologic differential diagnosis

The histologic differential diagnosis of Sweet's syndrome includes conditions microscopically characterized by either neutrophilic dermatosis or neutrophilic panniculitis (Table 9) [2-4,6,12,193,346-353]. Neutrophilic dermatoses include abscess or cellulitis, bowel (intestinal) bypass syndrome, erythema elevatum diutinum, granuloma faciale, halogenoderma, leukocytoclastic vasculitis, neutrophilic eccrine hidradenitis, pyoderma gangrenosum, and rheumatoid neutrophilic dermatitis. Culture of lesional tissue for bacteria, fungi, and mycobacteria should be considered to rule out infection since the pathologic changes associated with Sweet's syndrome are similar to those observed in an abscess or cellulitis [23].

**Table 9:** Histologic differential diagnosis of Sweet's syndrome

Abscess/cellulitis	Positive culture for infectious agent
Bowel (intestinal) bypass syndrome	History of jejunal-ileal bypass surgery for morbid obesity
Erythema elevatum diutinum	Erythematous asymptomatic plaques often located on the dorsal hands and elbows; younger lesions have microscopic features of leukocytoclastic vasculitis, whereas older lesions have dermal fibrosis and mucin
Granuloma faciale	Yellow to red to brown indurated asymptomatic facial plaques; there is a grenz zone of normal papillary dermis beneath which there is a dense diffuse inflammatory infiltrate of predominantly neutrophils (with microscopic features of leukocytoclastic vasculitis) and frequently numerous eosinophils
Halogenoderma	Neutrophilic dermal infiltrate with necrosis and pseudoepitheliomatous hyperplasia with intraepidermal abscesses; history of ingestion of bromides (leg lesions), iodides (facial lesions), or topical fluoride gel to teeth during tumor radiation therapy to face
Leukemia cutis	Dermal infiltrate consists of immature neutrophils
Leukocytoclastic vasculitis	Vessel wall destruction – extravasated erythrocytes, fibrinoid necrosis of vessel walls, karyorrhexis, and neutrophils in the vessel wall
Lobular neutrophilic panniculitides	In addition to subcutaneous Sweet's syndrome, these include alpha 1-antitrypsin deficiency syndrome, factitial panniculitis (secondary to the presence of iatrogenic or self-induced foreign bodies), infectious panniculitis (secondary to either a bacterial, fungal, mycobacterial, or protozoan organism), pancreatic panniculitis, rheumatoid arthritis-associated panniculitis
Neutrophilic eccrine hidradenitis	Neutrophils around eccrine glands, often in patients with acute myelogenous leukemia receiving induction chemotherapy
Pyoderma gangrenosum	Painful ulcer with overhanging, undermined violaceous edges
Rheumatoid neutrophilic dermatitis	History of rheumatoid arthritis, nodules, and plaques
Sweet's syndrome	Acute onset, fever, neutrophilia, and painful plaques

Source **[346]**: Adapted with permission from Cohen PR: Paraneoplastic dermatopathology: cutaneous paraneoplastic syndromes. Adv Dermatol 1995;11:215–252. *Copyright 1995, Reprinted with permission from Elsevier Ltd, Oxford, United Kingdom*.

Leukemia cutis can occur concurrently with Sweet's syndrome. It can also mimic the dermal changes of Sweet's syndrome. However, in contrast to the mature polymorphonuclear neutrophils found in Sweet's syndrome, the dermal infiltrate in leukemia cutis consists of malignant immature leukocytes [354].

Subcutaneous Sweet's syndrome lesions may have pathologic changes in the adipose tissue that can be found in either the lobules, the septae, or both. Hence, the adipose tissue changes of subcutaneous Sweet's syndrome are similar to those of other conditions characterized by a neutrophilic lobular and/or septal panniculitis. Therefore, alpha 1-antitrypsin deficiency, factitial panniculitis, infection, leukocytoclastic vasculitis, pancreatitis, and rheumatoid arthritis should be considered and ruled out [2,4].

## Management

Sweet's syndrome lesions, if untreated, can remain for weeks to months. However, without any therapeutic intervention, the dermatosis-related symptoms and cutaneous lesions eventually resolved in some patients with classical Sweet's syndrome [10]. Cure or remission of the dermatosis-related cancer in patients with malignancy-associated Sweet's syndrome is occasionally followed by resolution of the individual's Sweet's syndrome. And, in patients with drug-induced Sweet's syndrome, spontaneous improvement and subsequent clearing of the syndrome occurs after stopping the associated medication. Surgical intervention has also occasionally promoted resolution of the patient's Sweet's syndrome when the dermatosis was associated with therapy amendable tonsillitis, solid tumors, or renal failure.

### Topical or intralesional corticosteroids

Topical or intralesional corticosteroids can be used to treat patients who have a small number of localized Sweet's syndrome lesions as either monotherapy or concurrently with another therapy [1,2,7]. High potency topical corticosteroids (such as 0.05% clobetasol propionate) in either a cream base, an ointment base, a gel base, or a foam base can be applied to the lesions [13,19-21,30,234,362-365]. Individual lesions have improved following a single injection or multiple intralesional treatments with triamcinolone acetonide when used at a dose ranging from 3 mg/ml to 10 mg/ml [362,366,367].

### First-line systemic agents

Systemic corticosteroids are the therapeutic mainstay for Sweet's syndrome. Other first-line systemic treatments for Sweet's syndrome are potassium iodide and colchicine (Table 10) [10,12,17,20,23,30,49,70,143,184,198,203,221,223,231,240,245,250,259,261,281,284,294,296,329,359-363,368-384,397,410].

**Table 10:** First-line systemic agents for Sweet's syndrome

***Corticosteroids***	
Prednisone	1 mg/kg/day (usually ranging from 30 mg to 60 mg) as a single oral morning dose. Within 4 to 6 weeks, taper dose to 10 mg/day; however, some patients may require 2 to 3 months of treatment or intravenous therapy [10,23,49,250]
Methylprednisolone sodium succinate	Intravenously administered (up to 1000 mg per day) over 1 or more hours, daily for 3 to 5 days. This is followed by a tapering oral dose of corticosteroid or another immunosuppressant agent [70,184,223,240,359-361].
***Potassium iodide***	Administered orally as 300 mg enteric-coated tablets, 3 times each day (for a daily dose of 900 mg) or as a saturated solution (1 gram/ml of water) of potassium iodide (SSKI, also referred to as Lugol's solution), beginning at a dose of 3 drops 3 times each day (9 drops/day = 450 mg per day) and increasing by 1 drop 3 times per day, typically to a final dose of 21 drops/day (1050 mg) to 30 drops/day (1500 mg) [17,20,23,143,198,361-363,368-374,397].^a^
***Colchicine***	Administered orally at a dose of 0.5 mg three times each day (for a daily dose of 1.5 mg) [20,30,281,284,329,360,371,373,375-377,410].

^a^1 drop = 0.05 ml (or 50 mg when the concentration of potassium iodide is 1000 mg/ml) when a "standard" medicine dropper (which dispenses 20 drops per ml) is used.

Source **[1]**: Adapted with permission from Cohen PR, Kurzrock R: Sweet's syndrome revisited: a review of disease concepts. Int J Dermatol 2003;42:761–778. *Copyright 2003, Reprinted with permission from the International Society of Dermatology, Blackwell Publishing Ltd, Oxford, United Kingdom*.

#### Corticosteroids

Systemic corticosteroids are the "gold standard" of therapy for Sweet's syndrome [7,8,10,12,16,17,19,20,23,36,49,50,70,184,223,233,240,250,284,358-361]. Dermatosis-associated symptoms improve promptly after treatment has been started and the cutaneous lesions resolve subsequently. Systemic corticosteroid therapy often begins with 1 mg/kg/day of prednisone as a single oral morning dose. Usually, the dose can be tapered to 10 mg/day within 4 to 6 weeks. However, some patients may require treatment for 2 to 3 months.

Intravenous corticosteroid therapy may be necessary in those Sweet's syndrome patients whose dermatosis has been refractory to other treatments [400]. Daily pulse intravenous methylprednisolone sodium succinate (at a dose of up to 1000 mg/day) over 1 or more hours for 3 to 5 days can be given. Upon conclusion of the course of intravenous treatment, either a tapering oral dose of corticosteroid or another immunosuppressant agent usually follows.

#### Potassium iodide

Horio *et al *[143] originally described the dramatic improvement in patients with Sweet's syndrome who were treated with potassium iodide in 1980. He confirmed his earlier observations with a larger study in 1983 [368]. Subsequently, several other investigators have also observed similar improvement when using potassium iodide to treat patients with Sweet's syndrome. Vasculitis and hypothyroidism are potential drug-induced side effects of potassium iodide [385].

After the initiation of potassium iodide therapy, symptoms of the dermatosis typically resolve within 1 to 2 days and skin lesions subside within 3 to 5 days. Potassium iodide, when available as a 300 mg enteric-coated tablet, can be administered orally 3 times each day (for a total daily dose of 900 mg). Alternatively, when the drug is available as a saturated solution (1 gram/ml of water) of potassium iodide (SSKI, which is also referred to as Lugol's solution), it is initially given at a dose of 3 drops 3 times each day. When a "standard" medicine dropper (which dispenses 20 drops per ml) is used, 1 drop equals 0.05 ml (or 50 mg when the concentration of potassium iodide is 1000 mg/ml). Therefore, the initial dose is 9 drops per day which equals 450 mg of potassium iodide per day. The dose is increased by 1drop 3 times each day, typically to a final dose between 21 drops per day (1050 mg) to 30 drops per day (1500 mg).

#### Colchicine

The efficacy of colchicine for Sweet's syndrome was initially reported by Suehisa and Tagami [373] in 1981. Two years later, in 1983, Suehisa *et al *[375] reported 3 additional patients with Sweet's syndrome who were successfully treated with colchicine. Colchicine may cause gastrointestinal symptoms such as diarrhea, abdominal pain, nausea and vomiting. These potential adverse effects from colchicine may improve after lowering the daily dose of the drug [2].

Several larger studies have subsequently confirmed that colchicine is an effective agent for the successful management of patients with Sweet's syndrome. For example, Maillard *et al *[329] presented 20 patients with Sweet's syndrome of whom 90% (18 individual) responded to colchicine therapy: fever resolved within 2 to 3 days, skin lesions attenuated within 2 to 5 days, arthralgia disappeared within 2 to 4 days, and leukocytosis normalized within 8 to 14 days. Similar to earlier studies, the starting dose of colchicine was 0.5 mg orally 3 times each day (for a total daily dose of 1.5 mg); treatment ranged from 10 to 21 days (mean = 15 days).

### Second-line systemic agents

Second-line agents for treating Sweet's syndrome include indomethacin, clofazimine, cyclosporin, and dapsone (Table 11) [1,12,17,20,30,203,221,231,245,259,261,284,294,296,372,378-384,421]. All of these agents have been used as monotherapy either in the initial management of the patient or after first-line therapies have failed. In addition, cyclosporine and dapsone have been used in combination therapy either with other drugs or as a corticosteroid-sparing agent [1,2,7,303,421].

**Table 11:** Second-line systemic agents for Sweet's syndrome

***Indomethacin***	Administered at a oral daily dose of 150 mg for 7 days, and then 100 mg per day for 14 days [259,261,284,378].
***Clofazimine***	Administered orally at a daily dose of 200 mg for 4 weeks, and then 100 mg per day for 4 weeks [12,296,379].
***Cyclosporin***	As monotherapy or as a second-line agent (after failure of first-line therapy or as a corticosteroid-sparing agent) [12,30,231,294,380,381]. Initial oral daily dose ranged from 2 mg/kg/d [380] to 4 mg/kg/d [231] to 10 mg/kg/d [12,381]; in the latter patient, from the 11th day the dose was reduced by 2 mg/kg/d every 2 days and discontinued on day 21 [12,381].
***Dapsone***	As either monotherapy or in combination therapy. Initial oral dose ranged from 100 mg per day to 200 mg per day; the latter dose was either administered as a single dose or divided into 2 equal doses [17,20,30,203,221,245,284,372,382-384,421].

Source **[1]**: Adapted with permission from Cohen PR, Kurzrock R: Sweet's syndrome revisited: a review of disease concepts. Int J Dermatol 2003;42:761–778. *Copyright 2003, Reprinted with permission from the International Society of Dermatology, Blackwell Publishing Ltd, Oxford, United Kingdom*.

#### Indomethacin and clofazimine

Indomethacin and clofazimine have each been described in individual case reports and a single larger study to be effective for the management of patients with Sweet's syndome. In 1997, Jeanfils *et al *[261] reported the therapeutic efficacy for 17 of the 18 patients with Sweet's syndrome who received indomethacin as first-line monotherapy: an oral daily dose of 150 mg for 7 days and then 100 mg per day for 14 days. Von den Driesch [12] reported "almost complete remission" in 6 patients who were treated with clofazimine. The patients had chronic and relapsing Sweet's syndrome and had previously been unsuccessfully treated with methylprednisolone; they received an oral daily dose of 200 mg of clofazimine for 4 weeks and then 100 mg per day for 4 more weeks. None of the 6 patients required systemic treatment of their Sweet's syndrome after the clofazimine was discontinued.

#### Cyclosporin and dapsone

Cyclosporin and dapsone have been used either as monotherapy or in combination with other agents. The initial oral dose of cyclosporin ranged from 2 mg/kg/day [380] to 10 mg/kg/day [12,381]; for the patient who was receiving 10 mg/kg/day, the dose was reduced by 2 mg/kg/day every 2 days and discontinued on day 21 [12,381]. The initial oral dose of dapsone ranged from 100 mg per day to 200 mg per day [17,20,30,203,221,245,284,372,382-384,421].

### Other systemic agents

There are individual case reports of patients with Sweet's syndrome whose dermatosis has improved after receiving systemic therapy with antibiotics [7]. For example, these include individuals whose lesions have become secondarily impetiginized with *Staphylococcus aureus*; their dermatosis-related skin lesions often partially improve after treatment with an antimicrobial agent to which the bacterial strain is susceptible [23]. The symptoms and lesion of Sweet's syndrome also resolved in other patients with inflammatory bowel disease (treated with metronidazole) [267,387], and persons with dermatosis-related *Yersinia *[125,126] or *Chlamydia *[306,307] infection (treated with either doxycycline [125,389], minocycline [30,126], or tetracycline [306,307,388]). Resolution of Sweet's syndrome has also been observed following treatment with other antibiotics such as ciprofloxacin, metronidazole, penicillin, or pyrimethamine and sulfonamide; some of these patients also had Sweet's syndrome-associated infections caused by either group D *Salmonella *[401], *Salmonella typhimurium*, *Streptococcus*, *Helicobacter pylori*, or *Toxoplasma*.

Other systemic drugs have also been effective for the treatment of Sweet's syndrome. These observations have predominantly been described in case reports. The agents include cytotoxic chemotherapies and antimetabolites (chlorambucil and cyclophosphamide) [30,39,148,200,251,360,390], immunoglobulin [303], interferon alpha [202,366], etretinate [361], and tumor necrosis factors antagonists (etanercept [392], infliximab [264,278,266], and thalidomide [5,393,425]). Pentoxifylline was postulated to be of therapeutic benefit for treating Sweet's syndrome [394,395]; however, it was not found to be efficacious when used as monotherapy [1,2,7,295,362].

## Prognosis

### Clinical course

In some patients with classical Sweet's syndrome, the symptoms and lesions of Sweet's syndrome eventually resolved without any therapeutic intervention. However, the lesions may persist for weeks to months [10,23,254,355]. Successful management of the cancer occasionally results in clearing of the related dermatosis in patients with malignancy-associated Sweet's syndrome [13,15,23]. Similarly, spontaneous improvement and subsequent resolution of the syndrome typically follows discontinuation of the associated medication in patients with drug-induced Sweet's syndrome [13,15,23]. In some of the Sweet's syndrome patients who had dermatosis-associated tonsillitis, solid tumors, or renal failure, surgical intervention resulted in the resolution of the dermatosis [1,2,19,315,356,357].

Sweet's syndrome may recur following either spontaneous remission or therapy-induced clinical resolution [10]. The duration of remission is variable between recurrent episodes of the dermatosis. In cancer patients, Sweet's syndrome recurrences are more common. Indeed, the reappearance of dermatosis-associated symptoms and lesions in an oncology patient may represent a paraneoplastic syndrome which is signaling the return of the previously treated malignancy [1,2,15,135].

### Complications

Patients with Sweet's syndrome can develop complications which are either directly related to the mucocutaneous lesions or indirectly related to the Sweet's syndrome-associated conditions or both. Antimicrobial therapy may be necessary if the skin lesions may become secondarily infected. Reappearance of the dermatosis may herald the unsuspected discovery that the cancer has recurred in patients with malignancy-associated Sweet's syndrome. Disease-specific treatment may be warranted for the systemic manifestations of Sweet's syndrome-related conditions such as inflammatory bowel disease, sarcoidosis and thyroid diseases.

## List of abbreviation

kg = kilogram

mg = milligrams

ml = milliliter

SSKI = saturated solution of potassium iodide

## Competing interests

The author(s) declare that they have no competing interests.

## Authors' contributions

PRC drafted the entire manuscript and gives his approval of the version to be published.
